# TheSNPpit—A High Performance Database System for Managing Large Scale SNP Data

**DOI:** 10.1371/journal.pone.0164043

**Published:** 2016-10-25

**Authors:** Eildert Groeneveld, Helmut Lichtenberg

**Affiliations:** Institute of Farm Animal Genetics, Friedrich-Loeffler-Institute, 31535 Mariensee, Germany; Cincinnati Children’s Hospital Medical Center, UNITED STATES

## Abstract

The fast development of high throughput genotyping has opened up new possibilities in genetics while at the same time producing considerable data handling issues. TheSNPpit is a database system for managing large amounts of multi panel SNP genotype data from any genotyping platform. With an increasing rate of genotyping in areas like animal and plant breeding as well as human genetics, already now hundreds of thousand of individuals need to be managed. While the common database design with one row per SNP can manage hundreds of samples this approach becomes progressively slower as the size of the data sets increase until it finally fails completely once tens or even hundreds of thousands of individuals need to be managed. TheSNPpit has implemented three ideas to also accomodate such large scale experiments: highly compressed vector storage in a relational database, set based data manipulation, and a very fast export written in C with Perl as the base for the framework and PostgreSQL as the database backend. Its novel subset system allows the creation of named subsets based on the filtering of SNP (based on major allele frequency, no-calls, and chromosomes) and manually applied sample and SNP lists at negligible storage costs, thus avoiding the issue of proliferating file copies. The named subsets are exported for down stream analysis. PLINK ped and map files are processed as in- and outputs. TheSNPpit allows management of different panel sizes in the same population of individuals when higher density panels replace previous lower density versions as it occurs in animal and plant breeding programs. A completely generalized procedure allows storage of phenotypes. TheSNPpit only occupies 2 bits for storing a single SNP implying a capacity of 4 mio SNPs per 1MB of disk storage. To investigate performance scaling, a database with more than 18.5 mio samples has been created with 3.4 trillion SNPs from 12 panels ranging from 1000 through 20 mio SNPs resulting in a database of 850GB. The import and export performance scales linearly with the number of SNPs and is largely independent of panel and database size. Import speed is around 6 mio SNPs/sec, export between 60 and 120 mio SNPs/sec. Being command line based, imports and exports can easily be integrated into pipelines. TheSNPpit is available under the Open Source GNU General Public License (GPL) Version 2.

## Introduction

High throughput single nucleotide polymorphism (SNP) genotyping is evolving at a staggering rate, developing into a powerful tool in genetic analyses in all areas of biology [1]. Although the potential of this technology is immense, it is also associated with serious data processing issues. Dropping genotyping costs and ever increasing marker densities have resulted in a huge increase in data volume. This leads to drastic increases in data processing times which can only partly be mitigated through faster, bigger, thus more expensive disks.

In dairy cattle for example, more than 1.2 mio genotyped animals had been registered in the US CDCB database [2] as of February 2016, adding tens of thousands each month. SNP usage is of course not limited to animal agriculture; instead they are used across all domains of life.

Apart from the initial raw data management issues, SNP data analysis workflows often include filtering SNPs, thereby creating genotype subsets for different purposes. Keeping track of a multitude of derived SNP datasets is possibly a bigger issue than disk space requirements. Therefore, it makes sense to organize data management through the use of a relational database, which provides SNP access to multiple users.

A number of database developments have tried to address some of the above mentioned issues [3–6]. However, one main shortcoming is the wide spread use of the ‘one SNP per row’ storage scheme which leads to huge storage requirements and slow export rates. Rios et al. [7] improved this scheme by storing one record per individual containing all genotypes together with each genotype position. Baron et al. [6] addressed the storage and processing speed issues arising from the ‘one SNP per row’ storage scheme by introducing genotype blocks, which hold 5000 SNPs in one string. Our proposal goes well beyond this, through a generalized and more efficient storage scheme together with the development of the genotype set concept, which substantially enhances data manipulation.

## Scope and Objectives

Being defined as a data repository, TheSNPpit does not have any data analysis features of its own, since excellent software packages like PLINK [8] are available. Instead, the functionality is restricted to tasks that are relevant to the domain of data storage and management including the interfacing to downstream data analysis software like PLINK, GenABLE [9] and many others. TheSNPpit fills an expanding gap in the workflow of genomics data processing: it is the repository accumulating SNP data as they arrive from the genotyping service.

Lack of standardization of SNP data formats and ensuing problems are well known and their pitfalls have been discussed by Nelson et al. and Nicolazzi et al. [10, 11]. Indeed the importance of normalization of genotyping cannot be overstressed. However, we felt that this problem was beyond the scope of our package.

To circumvent this issue it was decided to use the quasi standard PLINK ped/map format [8] for data input, which can be produced by each genotyping platform. In itself, using PLINK format does in no way ensure proper integration of SNP data that might accumulate over time and get analyzed jointly as the data flow continues. As we require that the user supplies both the genotype data in the form of ped PLINK format as well as the annotation in the form of PLINK map files for each database import, it is clear that only compatible annotation and genotype files can be loaded.

Any researcher that does data analysis of genomic data in such a context has to be aware of the integration/annotation problem, thus, our defined starting point of PLINK ped/map data does not add a new level of complexity or uncertainty.

There are no restrictions for panel sizes, allowing their free mixture in the database. With this pre-condition data from different genotyping platforms—each with their own annotation—can be dealt with in one database.

The objectives for the development of TheSNPpit were:

1. Proliferating copies of the original SNP data, which are produced through filtering processes, will sooner or later lead to management and disk space issues. TheSNPpit should avoid the storage of multiple copies of nearly identical datasets: after their use the files can be removed, as they are easily and quickly restorable from the database. Obviously, such export/analyze/delete cycles are only a practical proposition if the exports are very fast.
2. TheSNPpit should be a SNP data storage system with an emphasis on management support. Consecutive subset definitions should be recorded automatically for documentation purposes.
3. SNP data files can be very large and indeed overwhelming both in terms of raw disk space as well as resulting processing time. With possibly millions of SNP genotype records from high density panels, both should be minimized.
4. Any biallelic system should be supported, such as AB or ATGC, which can be recovered on export exactly as previously imported.
5. Often phenotypes are recorded together with the SNP data. TheSNPpit should be sufficiently general to cover all SNP use cases, no matter from what organism the data is derived. Thus, no phenotypes can be implemented that are specific to certain use cases. Instead, a generalized phenotype system, which allows storage and retrieval of any phenotype attached to a sample or individual is required.
6. TheSNPpit should use a generalized internal format for SNP storage, thereby allowing import from any source.
7. The database must scale to allow storage of SNP data on millions of samples and different panel sizes, including SNPs from whole genome scans.
8. A command line interface aids the easy inclusion of TheSNPpit into workflows, following the UNIX paradigm of one program performing a small, accurately defined task and doing it well.
9. Solely open source software (OSS) including the operating system should be used to allow for widespread use and encourage adaption and further development by interested parties.

## Design: a fresh view

In their contribution Groeneveld and Truong [12] presented the basic ideas for an efficient SNP database system. The implementation was done completely in Perl without considering maximum performance and functional completeness. Instead, it served as a proof of concept, which clearly demonstrated the superiority in terms of processing speed and disk storage efficiency.

For a better understanding of the following implementation, its principles will be repeated briefly.

For a given SNP panel, a fixed number of SNPs is always genotyped for every sample. Each SNP is named—if known—and placed on the chromosome by base pair position and chromosome number. Accordingly, each SNP is usually considered an individual entity which is modeled accordingly in the database design through an instance of an entity with the genotype or its alleles being the only net data. For one plate with 96 wells for a 800K panel more than 76.8 million rows are created. Adding the keys for sample identification and SNP names to the genotypes easily leads to hundreds of MB to store just data from one plate in a relational database if SNP data are modeled as one SNP per row.

### View SNP from one sample as a vector

Alternatively, all SNPs can be viewed as elements of a vector, whose description is identical for each sample. As a result, no SNP-specific information needs to be stored with the genotype data, as the map is stored only once and the relevant SNP information can be derived from the SNP’s vector position. Thus, for the above mentioned plate only one record will be stored instead of the 76.8 mio. Obviously, each will be much longer: with an 800K panel it will consist of 1600K bytes, if the alleles are stored as one ASCII character each.

In biallelic systems only three states exist: the two homozygous genotypes for allele 1 and 2 and the heterozygous genotype. This requires only 2 bits, which can even accommodate no-calls or missing values. The genotype record can thus be viewed as a bit vector: the 800K record can now be stored in 1600K bits i.e. 196KB. More general, one byte can store 4 SNP genotypes, which amounts to 4.096 mio SNP for one MB.

### Definition of genotype sets

Genotype data are usually produced by genotyping labs in batches and need to be stored as they are delivered to the user. For analysis, on the other hand, often a data set comprising certain SNPs from a defined set of samples is required. Thus, the import or loading of new data will happen in smaller or larger batches, while the extraction or export should allow specification of SNPs and samples.

This is achieved through two selection vectors *individual_selection* and *snp_selection*. The former is a list of sample IDs used for locating the genotype vector of certain samples. The latter has the dimension of the panel size with a 1 in the SNP position to be extracted. Both vectors are used in the export phase: retrieval of the compressed genotype vectors is determined through the entries in the individual selection vector, while the SNP selection vector determines which SNPs are to be written to the export vector. For repeated use of a particular genotype set—be it as the basis for a new subset definition or for export—only the two vectors need to be stored in the database and be given a name: the exports can be executed solely on the basis of the genotype set name [12].

The storage space requirements for a genotype set in the database are minimal: the 800K panel requires an equal number of entries, 800K, in a bit array, amounting to 97.7KB, while the individual selection vector only has the length of the number of samples in the export set.

### Definition of phenotypes

Sometimes phenotypes are recorded along with genotype data. We have implemented a generalized mechanism for storing additional data for each sample which can be freely defined by the user.

## Implementation

TheSNPpit has been written exclusively using open source software and is distributed under the GNU General Public License (GPL) Version 2 or newer [13]. The framework is written in Perl, while PostgreSQL [14] serves as a database backend. The computationally intensive tasks for packing and unpacking the individual SNPs are implemented in C using the ECPG library for database access. Thus, any up-to-date Linux system should be able to execute the software.

**Fig 1:**
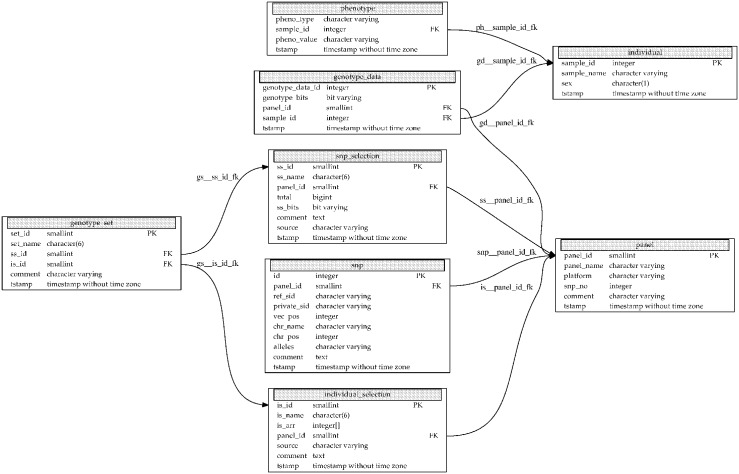
Entity relationship diagram of TheSNPpit. Each box represents a table and each entry within a box a column in the respective table together with its data type. Primary (PK) and foreign keys (FK) are marked with their abbreviations and arrows.

Fig 1 presents the entity relationship diagram of TheSNPpit. The entity which possibly creates large data volumes is column *genotype_bits* in table *genotype_data*. This vector uses 2 bits per SNP for storing the four states: homozygous for allele 1, homozygous for allele 2, heterozygous and the no-call, as described above. In PostgreSQL only a 4 byte overhead is incurred for each such bit array, with no practical limitations to its length, which has been tested for panel sizes up to 50 mio SNPs.

As described in more detail below in Listing 4, a SNP and an individual/sample selection vector define a genotype set. The SNP selection vector is implemented as a ‘bit varying’ data type, using one bit for each SNP in the *genotype_bits* vector. Thus, for an 800K SNP record only 25KB are needed. The individual selection vector is implemented as an integer array, storing the integer keys of the *sample_id* from table *individual*. Even with possibly tens of thousands of samples, this vector requires relatively little storage space, in the order of kilo- to megabytes.

Defining a new subset of the originally loaded SNP data thus amounts to only storing a new SNP and individual selection vector in tables *snp_selection* and *individual_selection* and one record in *genotype_set* resulting in a few KB or perhaps MB. This is the reason why subsets can be defined at virtually no storage costs.

Timestamps are added to all records in the database, which gives the user the opportunity to follow up on certain batches of data.

All functions are implemented in the standard UNIX format as command line instructions with a number of options. This facilitates an integration of TheSNPpit actions into genomic pipelines and workflows.

## Functionality

TheSNPpit is intended to be a highly efficient database for storing, defining subsets and extracting SNP data. Accordingly, its features are limited to actions required for this scope. The program can either be invoked as ‘TheSNPpit’ or ‘snppit’ for practical reasons. The main functions are:

**Figure:**
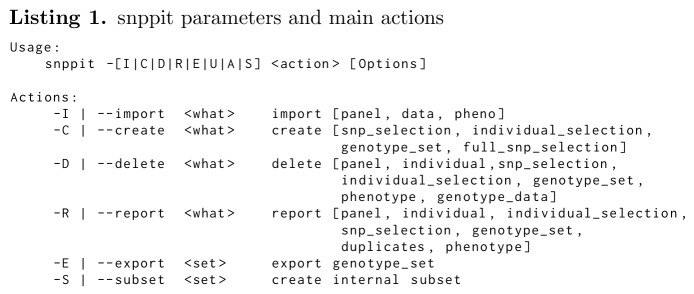


### Importing data

This function inserts new panels as well as SNP data (see Listing 2). Standard PLINK maps and peds are processed [8]. SNP data can only be entered relative to previously loaded map data, thus map files need to be imported first.

**Figure:**
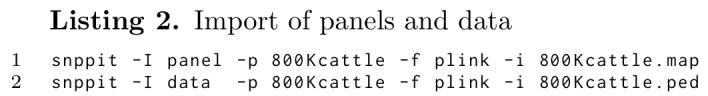


The standard map file consists of the columns: chromosome name/number, SNP name, position, and possibly the biallelic variant for the SNP, such as AB or AT GC. The latter is not part of the PLINK map file format but was added to support storage of alleles and their recovery upon exports.

For each data import a standard PLINK ped and map [8] file is required to ensure unambiguous SNP mapping.

### Creating subsets

The definition of subsets and/or genotype sets is a strong feature of TheSNPpit.

**Figure:**
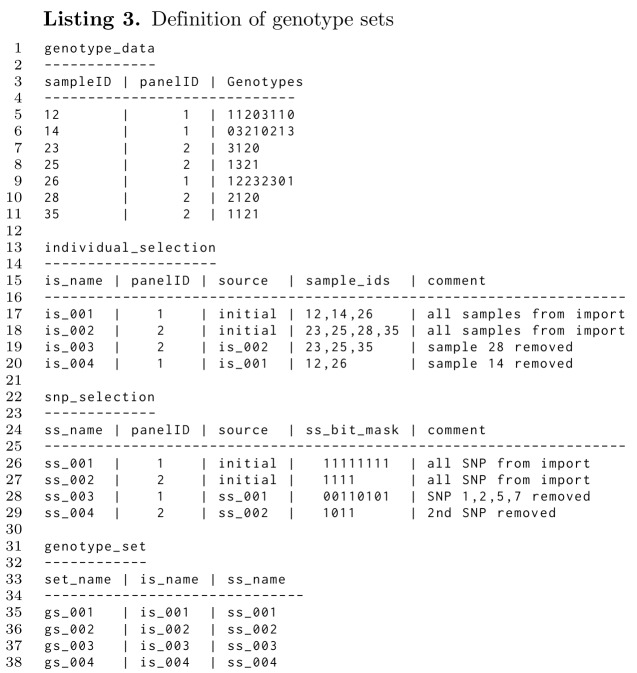


In Listing 3 the process of defining subsets is explained. Initially, SNP data from two panels are loaded resulting in the *genotype_data* table with content as given in line 5-11 with 8 SNP for panel 1 and 4 SNP for panel 2. The genotypes are 0, 1, 2, 3 standing for homozygous 1, heterozygous, no call and homozygous 2. The loading process, with a function call similar to line 2 in Listing 2, not only loads the SNP data, but also creates an individual selection vector (Listing 3, line 17 and 18) and a SNP selection vector (Listing 3, line 26 and 27).

Next, we assume that we want to define a subset for panel 1 which only includes SNPs 3, 4, 6, and 8 for samples 23, 25, and 35. The selection of individuals is reflected in a new vector *is_003*, as shown in Listing 3, line 19. The selected samples are loaded from the file *is_selection1.txt*, shown in line 1 of Listing 4. The SNP selection is specified through a SNP selection vector (*ss_003* in Listing 3, line 28) loaded from a file *ss_selection1.txt* (Listing 4, line 5). Those SNPs that are not part of the new subset are identified through a 0.

As the last step, a new genotype set *gs_003* is defined by inserting a new line in table *genotype_set* as shown in line 37 of Listing 3. Through the name of the genotype set its SNPs and individuals are uniquely defined, which is used for exporting and further subset definition.

**Figure:**
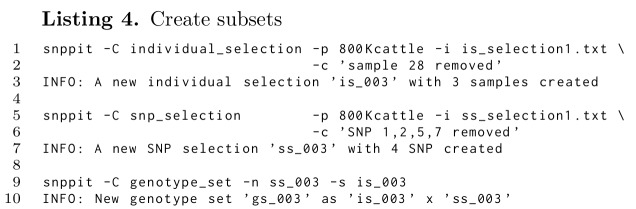


Apart from explicitly defining selection vectors based on sources external to the database, as shown in Listing 4 and through the initial data import (Listing 3) these two selection vectors can also be created within the database on the basis of filters based on major allele frequencies (maf), frequencies of no-calls for samples and SNP, and chromosomes. Each such filtering can create a new genotype set, which can be the basis for subsequent filtering. An example is given in Listing 5.

Line 1 through 5 of Listing 5 are subset operations executed in the database after the initial data have been imported into an empty database, which resulted in the genotype set *gs_001*. Line 13 states that *gs_001* is based on *is_001* and *ss_001*, while lines 26 and 38 give further details on those two vectors: line 26 provides the information on the panel size (580961 SNPs), and line 38 states that 5498 samples are involved.

The input data (*gs_001*) included duplicates, which are removed in line 1, thus creating a new genotype set *gs_002*. This genotype set, *gs_002*, uses the full SNP selection vector, but the smaller *is_002*, which has 11 fewer entries than the original *is_001*, as seen in lines 14 and 39.

In line 2 a new set is created which does not include the W chromosome, leading to *gs_003*, which is based on a new SNP selection vector *ss_002* with all SNPs on chromosome W set to 0. The details are given in line 27: the number of SNPs is now 580947, 14 fewer than those from *ss_001* in line 26. The indention shows the hierarchical definition as *ss_002* being based on *ss_001*, with the source being explicitly given in the *Source* column.

Line 3 now filters low quality samples and SNPs by setting the no-call frequency threshold to .05 and that for the SNPs to .03 based on the genotype set *gs_003*, which is the result of the the subset definition in lines 1 and 2. This results in a modification of number of active SNPs and a reduced number of samples (line 16, 29, and 40).

Lines 4 and 5 from Listing 5 finally each add a filter on the basis of major allele frequencies, first at .03 and then additionally at .05 based on the set from the .03 filter. As before, the SNP and individual selection lists provide the detailed outcome of these filtering steps.

The format chosen should help to keep track of subsets through the indention and the *Source* information.

**Figure:**
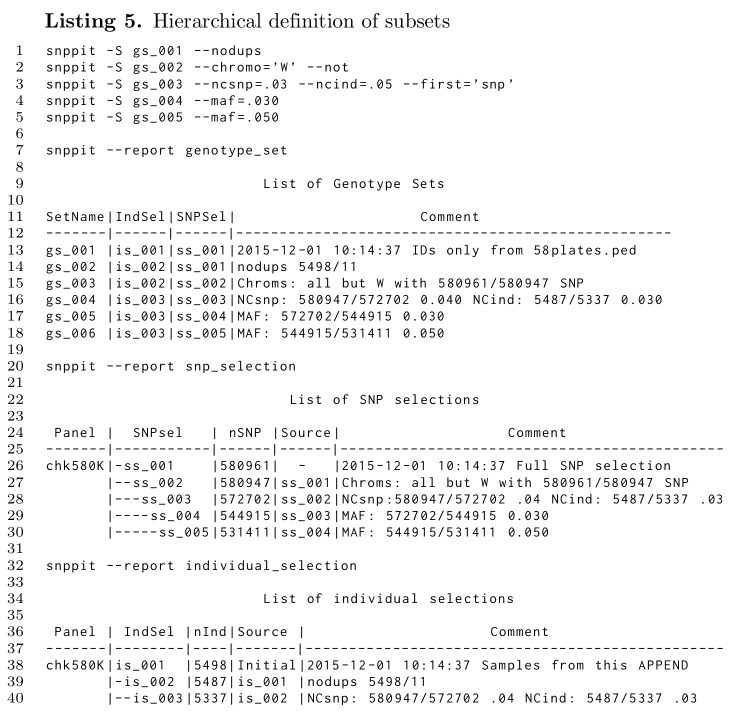


### Phenotypes

Often phenotypes are recorded along with genomic data. For a generalized approach we have implemented an entity-attribute-value model. Usually phenotype traits are column names in the database structure. This is appropriate, when a well defined set of phenotypes is to be stored. However, the TheSNPpit developers do not know, what phenotypes the users will want to store. This functionality allows the user to specify phenotype names and values as part of the data to be loaded. The phenotypes are linked to the sample name with no restriction in the number of different phenotypes. Upon export, the database is scanned for phenotypes from those samples included in the export. If found, they are exported as a csv file for further processing, one line for each sample with the sample ID, the phenotype names and data across.

**Figure:**
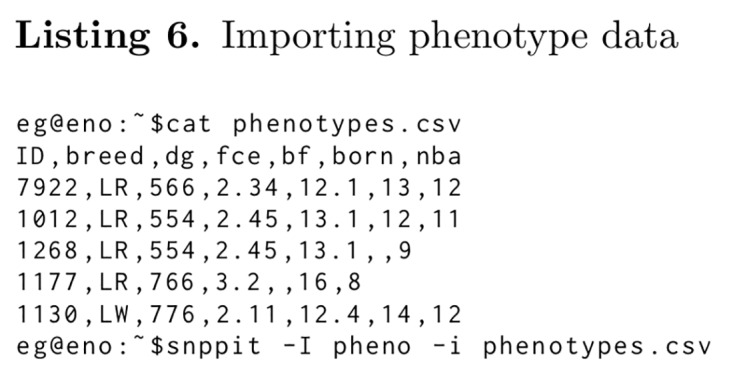


Listing 6 gives an example of the phenotype input file and the snppit command to import it. The first column is the sample ID, followed by the phenotype names. The following lines show the actual phenotypes for each sample ID, all values in standard comma separated value (csv) format.

### Export

Being a SNP repository, exports of previously defined genotype sets are the main activities of TheSNPpit. The philosophy is: define a subset in TheSNPpit, export, use and delete it. If this file needs to be used again, then a very fast export will provide the data file. The default output format is PLINK: each export produces one map and one ped file [8]. The “0125” is an additional format with the digits indicating homozygous for allele 1, heterozygous, homozygous for allele 2, and missing. Both output formats are available, independent of what data format was initially imported. The output files can be quite large, for instance 20000 700K records produce a file size of 52GB. Here a slow hard drive can easily be the speed limiting factor, which is particularly true for network connected drives.

Listing 7 shows the pseudocode of how subsets are actually created from the genotype set names and their defining SNPs and individual selection vectors. As can be seen, the database is only accessed once to recover all SNPs for a sample independently of the panel’s size: be it 8 SNPs as used in the example or millions from high density panels or even whole genome sequences.

**Figure:**
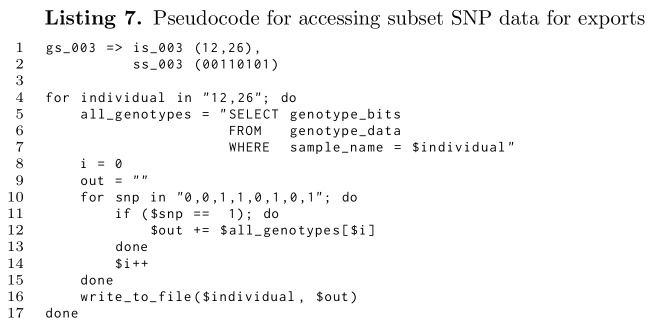


### Reporting

For housekeeping and database management, a number of reports can be generated. The listings provide information on the database size, size of panels and the number of SNPs stored. Furthermore, each record is tagged by a timestamp at database insertion. Samples with more than one genotype record can be listed. Content of specified selection vectors as well as phenotypes of a given genotype set can be produced. Also, SNP names and sample IDs can be exported for given subsets. To keep track of subsets, the user can specify comments. If chosen prudently, these comments will help to document database actions as part of the database content.

### Delete and update

A number of delete and update functions have been implemented that should be sufficient for normal operations. Deletes can be made for all major entries like panel, individual, genotype sets, individual and SNP selection vectors, and genotype records. Depending on what is to be deleted cascading deletes will be performed.

## Benchmarking

TheSNPpit design goals were high execution speed, great storage efficiency and scaling to very large databases. In the following we shall investigate the scaling of TheSNPpit with respect to panel sizes for import and exports for various sets and subsets of SNP data. All timings where obtained on an Ubuntu system with 16GB of RAM and a quad core Intel i5-4670 CPU and recorded as wall clock time. The timings include only the actual inserts and exports and not the initial reading of the map information from the database at start up, which would unduly punish genotype sets with small numbers of records.

Synthetic SNP records were generated for various panel sizes to investigate scaling and performance.

### Import

**Fig 2:**
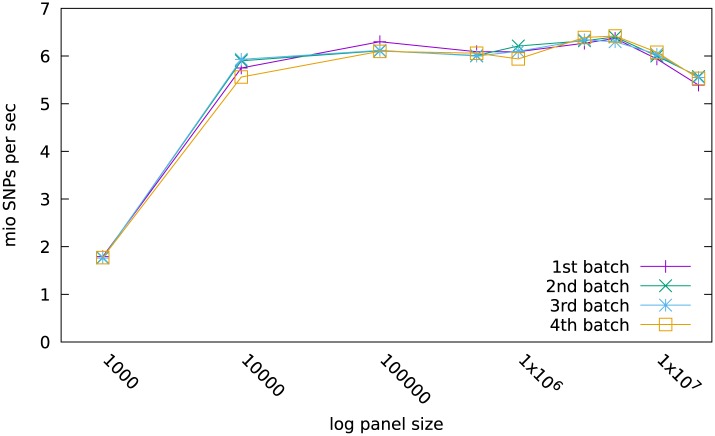
Performance of imports. Performance in mio SNPs/sec of consecutive imports of 4 batches of 100 mio SNPs from 111368 samples typed on panels ranging from 1000 to 20 mio.

Fig 2 shows the import performance of SNPs from various panel sizes, ranging from 1K to 20000K, all of which contained a total of 100 mio SNPs. For the 1K panel 100000 samples were loaded while only 5 records made up the 100 mio SNPs for the 20 mio panel size. In reality, most panel sizes currently go up to 1000K or perhaps 3000K. Here, the two largest panels with 10 and 20 mio SNPs, respectively, were used to see if SNP panels derived from whole genome sequencing, which are in the order of tens of millions, could also be handled.

In the first run 111368 samples with a genotype record and 900 mio SNPs were loaded into the empty database. This process was repeated 4 times resulting in batches of 445472 samples with a total of 3600 mio SNPs to test the scaling of import speed as the database fills. The import performance was measured as million SNPs per second and plotted separately for each batch (Fig 2).

Loading the 100000 individuals with the small 1K panel incurred a substantial overhead, resulting in a performance of just below 2 mio SNPs per second. But already from the 10K panel on, the performance is basically constant at around 6 mio SNPs per second. Thus, loading 100 mio SNPs takes only around 16 seconds. Only when records from the 10 and 20 mio panel were imported, a small reduction down to 5.5 mio/sec was incurred.

Thus, the import rate is nearly independent of the panel size across the vast range of 10K to 20000K. Therefore, SNP records derived from whole genome sequences can be handled just as well as regular low and high density SNP arrays.

Furthermore, the import rate speed is also independent of the fill of the database over the range investigated.

### Export

Repeated export-analysis-delete cycles of SNP data, to avoid subset file accumulation, are only feasible, if exports are no bottle necks. In databases, which store single SNPs recordwise exports can easily take hours.

Benchmarking the exports will provide an answer if the highly optimized C implementation does indeed facilitate repeated export-analysis-delete cycles of SNP data.

#### Exports of full genotype sets

**Fig 3:**
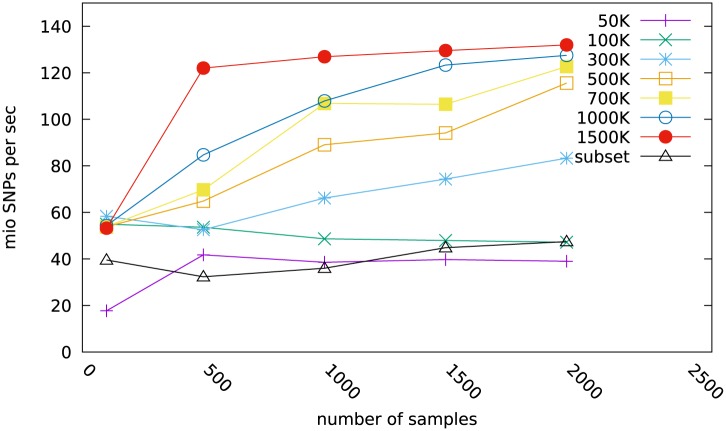
Performance of exports. Performance in mio SNPs/sec of exports of full genotype sets ranging from 100 to 2000 samples typed on panel ranging from 50K to 1.5 mio SNPs. The ‘subset’ is an export of a reduced genotype sets with 233319 SNP based on the selection of chromosomes 1, 3, 5, 12, 17, 25, and 29 from the original 1500K.

Fig 3 shows timings for exporting genotype sets of sample numbers ranging from 100 to 2000. The panel sizes ranged from 50K to 1500K. The exports were conducted from a database which consisted of 35700 samples with a total of 21.165 billion SNP.

It can be observed that the speed of export increases with both panel size and number of samples in the export set. Export speeds for the 50K panels were around 40 mio SNPs per second, going up to more than 120 mio SNPs for the larger panels. It should be noted that exporting the 2000 sample 700K genotype set took only 15 sec wall clock time for the 1.4 billion SNP.

The lines for the 50K and 100K genotype sets do not show the increase in export performance that the larger panel sizes exhibit (Fig 3). However, the export of the 100mio SNPs from the 1000 samples from the 100K panel took only 2 sec which is very short for timing measurement. In multi user/multi tasking operating system environment some variation is likely to be expected.

#### Exports of derived and reduced genotype sets

Filtering on the basis of no-calls and major allele frequencies or exclusion of certain chromosomes leads to new subsets of genotypes. To assess the export speed of subsets and to quantify the performance penalty when a subset is exported relative to the full set, we created new SNP selections on the basis of chromosomes 1, 3, 5, 12, 17, 25, and 29 totalling 233319 SNPs, instead of the original 1.5 million. We tested genotype sets with 100, 500, 1000, 1500, and 2000 samples for a 1500K panel, but with only 233319 active SNPs each.

To be able to export the reduced SNP set, the complete 1500K packed genotype record is transferred from the database to the client program, where those indicated by the SNP selection vector are decoded. These are then copied to the output vector, which is finally written to the export file.

As can be seen from the bottom line (subset) in Fig 3 this export, basically of a 233K panel, approaches the export rate of the 100K, crossing its rate at 2000 samples. From this we conclude that the overhead to read and process a large panel to obtain a subset is small. As the proportion of SNPs exported from a full panel gets smaller, so does the export rate: during export, the SNP selection vector is scanned from beginning to end (see Listing 7). Therefore it is immaterial if a subset was defined on the basis of chromosomes, the no-call rate or a major allele frequency threshold. The subset will always export faster than the full set.

In absolute terms, exporting the 3000mio SNPs from the 2000 sample/1.5 mio genotype in this benchmark took only 25 sec.

### Database size

Highly compressed storage must be met with a large capacity to handle massive data in a database. This was tested by importing SNP data from more than 18 million samples of various panel sizes ranging from 1K through 20000K. The resultant database stored the 3.4 trillion SNPs in 840GB for the genotype data plus around 9GB for everything else including SNP names and sample IDs as shown in Listing 8. The 868 genotype sets created during the import phase used a mere 216KB, Listing 8, line 28, supporting the statement that derived genotype sets can be stored at nearly no costs.

**Figure:**
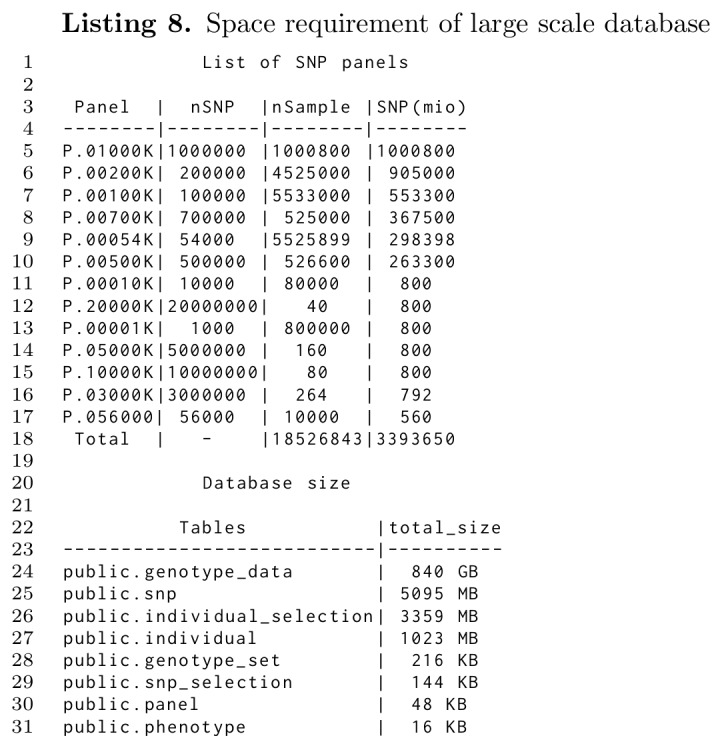


The export rate was independent of the database size. For a very large database with millions of samples, only the initial startup time may take some seconds longer. The ensuing export speed will then be identical in large and small databases. Thus, we conclude that massive databases with efficient imports and exports are possible with TheSNPpit, well beyond the here tested 850GB.

### Limitations

TheSNPpit software does not have inherent capacity limitations, but external factors may have effects. These can be the inherent limits of the file system used, the memory available and limitations of PostgreSQL. Clearly, with increasing panel sizes, more RAM will be required. With 64bit machines, the RAM will be no problem as only one genotype vector is handled at a time.

As of PostgreSQL-9.4 the maximum database size is 32TB with a limit of 1GB for a field. The 32TB will rarely be a limitation given our compressed SNP storage. The 1GB limit could conceivably only be reached by the packed *genotype_bits* in *genotype_data* as it increases with panel size. However, with our compressed storage a panel size of 4,294,967,290 SNPs could be handled by PostgreSQL in its 1GB, a limit that even data from whole genome scans will not approach.

## Discussion and Conclusions

TheSNPpit is a fast database system for storage and management of large volumes of SNP data. It can handle panels of any size, even those derived from whole genome scans. Through its compressed storage of genotypes, very large numbers of SNPs can be stored and exported efficiently. However, there are a few other software packages available that could possibly also be used for the intended purpose of TheSNPpit. SNPpy was already published in 2011 with an update in 2013 [5, 15], while dbVOR is much more recent from 2015 [6]. The design of the packages is quite different from TheSNPpit. Both have been developed to serve local data processing requirement. While SNPpy and dbVOR allow the storage of SNP data they do also have other capabilities that we did not consider essential for our target group of users.

In Table 1 we have compared the performance of TheSNPpit with dbVOR and SNPpy on import and export of SNP data. For comparable results we installed the latter two on the same computer that was used for the benchmarking of TheSNPpit reported above. We have expanded the performance comparison from Baron et al. (Table 5 in [6]) with a TheSNPpit column and added a larger dataset with 3.2 billion SNPs—which is closer to current day dimensions—to the original 139 mio SNP HapMap dataset. In line with [6] we present the timings from SNPpy when run in the single processor mode. All runs were done on an empty database. The timings were averaged over three runs for the first and two for the second dataset. Technically, we followed the excellent description of the ‘Performance Measurement Details’ provided by Baron et al. [16]. The results are presented in Table 1.

**Table 1:** Benchmark of three SNP database systems. Each dataset is loaded into an empty database. The timings are wall clock times averaged over three runs for the HapMap and two runs for the second data set.

Tasks	units	dbVOR	SNPpy	TheSNPpit
load all HapMap data 225@620K	h:m:s	00:09:08	00:10:38	00:00:22
export all genotypes to map/ped	h:m:s	00:04:59	00:04:04	00:00:07
export chromosome 1 to map/ped	h:m:s	00:00:25	00:00:42	00:00:07
database size	MB	522	9,086	73
load all data 5509@581K	h:m:s	01:10:04	03:58.06	00:10:14
export all genotypes to map/ped	h:m:s	02:58:04	01:29:16	00:01:16
export chromosome 1 to map/ped	h:m:s	00:22:37	00:30:21	00:00:19
database size	MB	8,198	201,326	868

In the 139 mio SNPs HapMap dataset, importing took around 10 minutes for dbVOR and SNPpy while TheSNPpit finished this task in 22 seconds. The exports of all 139 mio genotypes took 4 to 5 minutes for dbVOR and SNPpy versus 7 seconds with TheSNPpit. When it comes to exporting SNPs from chromosome 1 only, the difference becomes smaller but is also much less relevant because of the much shorter absolute time.

The more realistic second dataset with its 3.2 billion SNPs gives a better understanding of the relevance of processing speed. Import times are not really that crucial because loading of data is done only once. But still, also here, the import in TheSNPpit was much faster, taking only 10 minutes while the users needed to wait 1 hour and 7 minutes with dbVOR and nearly 4 hours when using SNPpy.

Exports are done more often, therefore, fast execution is more important. The excution timings are around 3 and 1.5 hours for dbVOR and SNPpy, respectively, while TheSNPpit finishes after 76 seconds.

When exports are done to a solid state drive (SSD) instead of the traditional hard disk drives (HD) the wallclock time for TheSNPpit halves going down to a mere 35 seconds, while the export time for dbVOR and SNPpy barely change.

It must be noted that SNPpy can make use of multiple CPUs. When using all four processors of our benchmarking system, the load time goes down from around 4 hours to less than 3 while the exports of all genotypes went down from 1.5 hours to 30 minutes. Still, TheSNPpy is much faster than either dbVOR and SNPpy.

While faster program execution is always nice to have, it allows or prevents some use cases once a certain limit is reached.

With exports of 3 hours the processing time will become unacceptably long. Users will rather keep copies over copies of derived files with genotype sets, instead of deleting and exporting them again when required. Thus, the high export speed not only reduces waiting time, but also allows—together with the novel genotype set system implemented in TheSNPpit—“export-analyze-delete” cycles, a very advanced approach to data management.

Another issue is scaling. For the HapMap dataset dbVOR exports the 139.5 mio SNPs at a rate of.47 mio/sec. On the larger second dataset (3200.5 million SNPs) this figure goes down to.30 mio/sec which constitutes a drop of 36%. The corresponding figures for SNPpy are.6 mio SNP per second for both datasets. TheSNPpit, on the other hand, exports the HapMap SNPs at 20.0 mio/sec while the second larger dataset gets exported at an even higher rate of 42.1 mio/sec on HD and 91 mio/sec on SSD. Thus, the export performance of dbVOR seems to deteriorate with larger datasets, SNPpy exports at the same rate, while TheSNPpit even increases its export speed going from a smaller to larger a dataset and that on a level that is two orders of magnitude higher.

Given today’s rate of genotyping the 5509 dataset is not really large. Based on the benchmark results an export for a 50000 sample dataset would require 30 hours of processing with dbVOR (assuming linear scaling) while the same job would be finished after 760 seconds with TheSNPpit and only 6 min when exporting to an SSD.

At the volume of such experiments, disk utilization becomes an issue. Here, the three database systems differ substantially. The SNPpy database grows to nearly 200GB for the larger dataset, while dbVOR uses 8GB and TheSNPpit not even 1GB. If used for the hypothetical 50000 sample high density dataset, the space requirement for SNPpy would increase to two TB.

While tens of thousends of samples will not be uncommon in one ‘experiment’, database will likely have to accommodate data from more sources, if centralized data management is considered important. If one wanted to use SNPpy instead of TheSNPpit for a database as given in Listing 8 its size would be in the order of 100TB. This does not sound practical even with today’s low disk storage costs. dbVOR will use much less disk space because of its more efficient storage scheme. However, it will still require close to 10 times more disk space than TheSNPpit.

Our conclusion is, that dbVOR and SNPpy are not suited for databases of this size, whereas TheSNPpit scales even to huge datasets as can be seen from Listing 8.

The high efficiency of TheSNPpit is to be predicted from our design, which has been abstracted to a high level of simplicity. The few lines in Listing 7 define the export operation for subsets of any proportions on any panel size. Using only one compressed genotype vector for each sample maximizes database transfer speed. In TheSNPpit costly SQL calls are replaced by bit masking vectors with high speed bit shifts implemented in C using pointer arithmetic for maximum speed.

## Future Developments

TheSNPpit uses ‘panel’ as a constituent component in its design for the definition of genotype sets. Low and high density panels in the database can contain the same SNP names. However, at this time, access to genotype data is strictly within panels. Implementing logical operations on genotype sets as indicated by Groeneveld and Truong [12] could provide an elegant way of merging genotype sets.

With the rapid development of whole genome sequencing the use of sequence data will become more prevalent. SNPs derived from VCF files can already now be stored in TheSNPpit without any problem. However, VCF file information cannot be handled at this stage, something that CanvasDB has been set up to handle [17]. Here, it may be useful to investigate ways of integrating SNP and sequence data.

Input formats other than PLINK map/ped may be desirable and could be added rather easily, as could further export formats such as the binary PLINK formats.

Command line interfaces (CLI) are common in bioinformatics as they support scripting which is highly advantageous in pipelines. However, some users might appreciate a graphical user interface (GUI) for a steeper learning curve. Both user groups would find their ideal interface, if a GUI and a CLI would coexist.

## Availability and Installation

An automated procedure has been developed for TheSNPpit installation on Debian/Ubuntu Linux machines (S1 File). All software components are freely available and installed by the *INSTALL* script on the target system under /usr/local/ with the constraint that PostgreSQL has to be of version 9.3 and up. Multiple users can be defined to interact with a database with layered access rights.

TheSNPpit is freely available under the GNU General Public License version 2.

A 65 page user’s guide is available. If interested contact the authors.

## Supporting Information

S1 File README.install. Installation information.
(INSTALL)
